# Schizophrenia Animal Modeling with Epidermal Growth Factor and Its Homologs: Their Connections to the Inflammatory Pathway and the Dopamine System

**DOI:** 10.3390/biom13020372

**Published:** 2023-02-15

**Authors:** Hidekazu Sotoyama, Hisaaki Namba, Manavu Tohmi, Hiroyuki Nawa

**Affiliations:** 1 Department of Molecular Neurobiology, Brain Research Institute, Niigata University, Niigata 951-8585, Japan; 2 Department of Physiology, School of Medicine, Niigata University, Niigata 951-8122, Japan; 3 Department of Physiological Sciences, School of Pharmaceutical Sciences, Wakayama Medical University, Wakayama 649-8156, Japan

**Keywords:** cytokine, neuregulin, inflammation, TLR, schizophrenia, ErbB kinase, negative symptom

## Abstract

Epidermal growth factor (EGF) and its homologs, such as neuregulins, bind to ErbB (Her) receptor kinases and regulate glial differentiation and dopaminergic/GABAergic maturation in the brain and are therefore implicated in schizophrenia neuropathology involving these cell abnormalities. In this review, we summarize the biological activities of the EGF family and its neuropathologic association with schizophrenia, mainly overviewing our previous model studies and the related articles. Transgenic mice as well as the rat/monkey models established by perinatal challenges of EGF or its homologs consistently exhibit various behavioral endophenotypes relevant to schizophrenia. In particular, post-pubertal elevation in baseline dopaminergic activity may illustrate the abnormal behaviors relevant to positive and negative symptoms as well as to the timing of this behavioral onset. With the given molecular interaction and transactivation of ErbB receptor kinases with Toll-like receptors (TLRs), EGF/ErbB signals are recruited by viral infection and inflammatory diseases such as COVID-19-mediated pneumonia and poxvirus-mediated fibroma and implicated in the immune–inflammatory hypothesis of schizophrenia. Finally, we also discuss the interaction of clozapine with ErbB receptor kinases as well as new antipsychotic development targeting these receptors.

## 1. Introduction

### 1.1. Biological Overviews of the Epidermal Growth Factor (EGF) Family

Prof. S. Cohen first purified EGF as an eyelid opening factor from the mouse submaximal gland with nerve growth factor (NGF) [1]. Subsequent studies verified that EGF is a potent cell growth factor for almost all epidermal cells and that hyper-signaling of EGF or EGF receptors is involved in cancer growth and metastasis [2,3]. EGF is abundant in the urine and amnion and is often referred to as urogastron [4]. NGF has become popular in developmental neuroscience with its marked trophic actions on the cell survival of neurons [5]. Although both EGF and NGF have the same historical and tissue origin, these factors have opened separate windows in new fields of science, leading to modern cancer biology and neurobiology, respectively.

**Figure 1:**
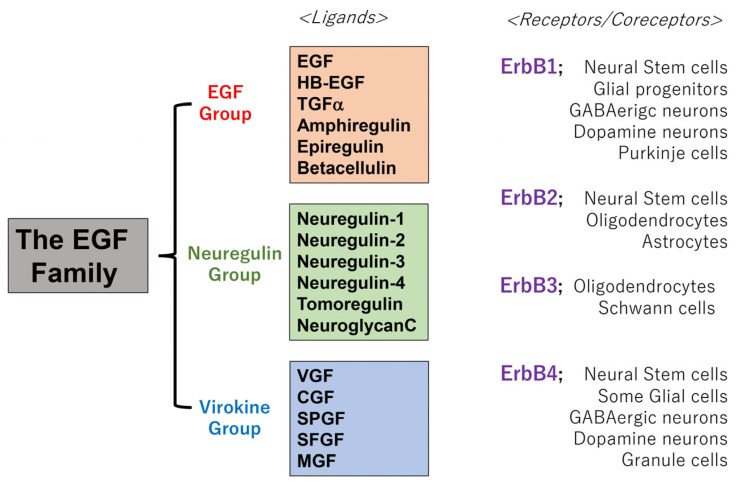
Groups and members of the EGF family and their receptors. Many EGF-like peptides structurally form the EGF family. These peptides are released from membrane-anchored precursor proteins via ectodomain shedding. These peptides bind to their receptor ErbB1-4 (Her 1-4) and induce intracellular signaling, although their interaction with ErbB2 is modest [16]. EGF receptor is abbreviated to EGFR alternatively. The localization of these receptors is shown in this figure. Abbreviations: EGF (epidermal growth factor), HB-EGF (heparin-binding EGF-like factor), TGFα (transforming growth factor alpha), VGF (vaccinia virus growth factor), CGF (cowpox growth factor), SPGF (smallpox growth factor), SFGF (shope fibroma virus growth factor), MGF (myxoma virus growth factor).

Molecular cloning and protein sequencing allowed us to discover novel homologs or orthologs of EGF, which can evoke the same or similar activities [6,7,8] and include three subfamilies: the EGF group, neuregulin group, and virokine group (Figure 1). Despite the peripheral functions of the EGF family in previous studies, the high expressions of heparin-binding EGF-like growth factor (HB-EGF) and transforming growth factor alpha (TGFα) have been reported in the central nervous system (CNS) [9,10]. High levels of neuregulin-1 mRNA and protein are also detectable in brain neurons [11,12], suggesting the hidden functions of the EGF family in brain development and plasticity [13,14,15].

One of the peculiar characteristics of EGF signaling is its role in viral proliferation [17,18]. The poxvirus family encodes an EGF-like molecule in its genome that promotes host expansion after infection. The EGF-like factors of poxvirus are called virokines, which share common core cysteine repeats and their surrounding amino acid sequences with the EGF sequence, reacting with the same receptors for the EGF family [17,18]. Similarly, the papillomavirus induces host cell proliferation and produces poxes, modifying EGF receptor signaling in host cells [19].

**Figure 2:**
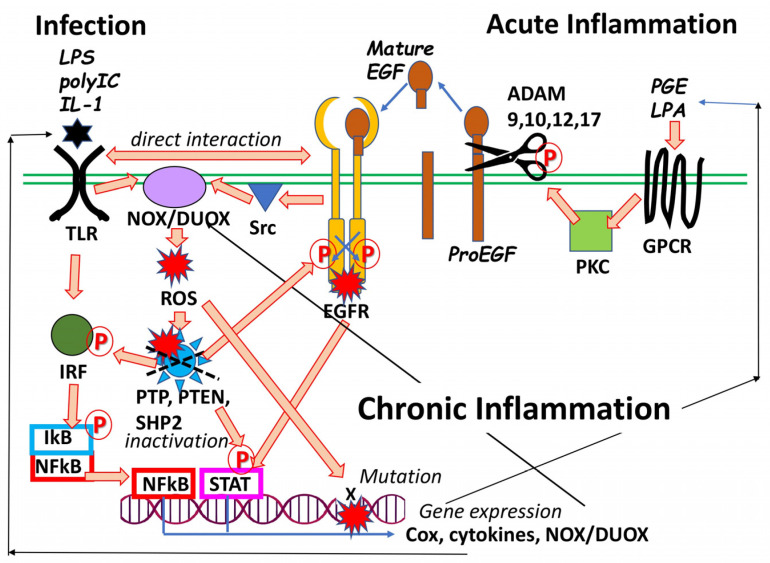
Interaction between Toll-like receptor (TLR) and EGF receptor tyrosine kinase (EGFR/ErbB1) in chronic inflammation and carcinogenesis. Many reports confirm the contribution of EGFR signaling to TLR-mediated chronic inflammation [37,38]. In addition, other studies indicate the requirement of EGFR in the TLR signaling itself [22,23,24]. EGFR signaling induces NADPH oxidase (NOX) activation resulting in the production of reactive oxygen species (ROS) and hydrogen peroxide, which inactivate protein phosphatases (SHP2, PTP1B, PTEN, etc.), elevating basal phosphorylation (P) or enhancing induced phosphorylation (P) of adaptor or signaling molecules: IRF, IkB, STAT, MAP, etc. Alternatively, ROS reacts with DNA, producing DNA mutation and leading to carcinogenesis. Elevation of their phosphorylation results in the DNA binding of NFkB, STAT, and AP-1 [39], leading to cell proliferation and gene expression of NOX, cyclooxygenase (COX), and cytokines and worsening inflammation. These promote the production of prostaglandins (PGE) and lysophosphatidic acid (LPA), which bind to their receptors of G-protein-coupled receptors (GPCRs). Activated protein kinase C (PKC) transactivates metalloproteases in a disintegrin and metalloprotease (ADAM) 9, 10, 12, or 17 [40,41]. ADAM cleaves the membrane-anchored EGF precursor or precursors of other members of the EGF family such as HB-EGF and TGFα and liberates mature EGF or its homolog, activating EGFR. These feed-forward and feed-back interactions between TLR and EGFR are implicated in the pathogenesis of chronic inflammation as well as in carcinogenesis.

Another peculiar feature of the EGF family has been elucidated in oncogenic biology as well as in immunology (Figure 2). It had been a mystery how tumorigenesis can be induced following chronic inflammation in the colon and lung [20,21]. EGF is one of the key molecules responsible for carcinogenesis resulting from chronic inflammation [20]. Toll-like receptor (TLR)-mediated or prostaglandin-triggered inflammation involves protein kinase C (PKC) signaling as well as ErbB kinase signaling [22,23,24]. The activation of PKC potentiates ADAM (a disintegrin and metalloprotease), eliciting ectodomain shedding of the EGF family’s precursors. The mature forms of active EGF homologs are released to stimulate proliferation of neighboring cells [25]. In turn, EGF/EGFR signaling in neighboring cells potently induces reactive oxygen species (ROS), activates STAT and NF-κB, and inactivates various phosphatases, leading to chronic inflammation [26] (Figure 2). Some reports propose direct molecular interactions between TLRs and ErbB receptor kinases as well [22,23,24]. These tight interactions and pathological connections between EGF signaling and the TLR system play a crucial role in current respiratory syncytial (RS)-virus-induced and COVID-19-induced cytokine storm and pneumonia [27,28]. Given the interactions between these systems, the EGF family and its signaling are augmented in the immune–inflammatory hypothesis of schizophrenia [29,30,31,32,33], potentially suggesting a substantial impact on the later risk of schizophrenia or other psychiatric diseases following the COVID-19 pandemic [34,35,36]. At present, there is no clear boundary between growth factors and inflammatory cytokines. Thus, members of the EGF family are currently classified into the category of cytokines as well.

Receptors of the EGF family are named ErbB1-4 or Her1-4 and belong to the receptor tyrosine kinase superfamily (Figure 1) [42,43]. Alternatively, EGF receptor is abbreviated to EGFR as well. A member of the EGF family binds to ErbB receptor kinases and evokes signal transduction via ErbB receptor homodimerization or heterodimerization [42,43]. In general, a ligand member in the EGF family binds to one of the ErbB1-4 receptors and recruits the second partner molecule from ErbB1-4 to induce their mutual trans-phosphorylation, evoking signal transduction from both ErbB molecules [43]. For example, EGF can evoke ErbB4 signaling by heterodimerization between the receptor of ErbB1 and the partner of ErbB4 [44].

As all neural stem cells are derived from the ectodermal layer, undifferentiated neural stem cells in the nervous system express high levels of ErbB1 or ErbB2 [45,46]. Following neural development and maturation, the level of ErbB1 is decreased, and ErbB4 expression is elevated, both of which are limited to midbrain dopaminergic neurons and GABAergic cells [46,47,48,49]. In the glial lineage, ErbB1 expression is replaced by ErbB3 during development. The interactions of neuregulins expressed on the neuronal surface with ErbB3 in oligodendrocytes promote their differentiation and myelination [50]. Accordingly, the EGF/neuregulin system contributes not only to the development of peripheral epidermal cells but also to the development and maturation of neurons and glial cells.

On the basis of these biological activities of the EGF family, we attempt to illustrate their neuropathologic contribution to schizophrenia as well as the phenotypic association of its model animals established by the members of the EGF family.

### 1.2. Phenotypic Association of The EGF Ligand Family with Schizophrenia

The association between this EGF family and schizophrenia was first reported in 2002 by two independent groups. We investigated neuropathological alterations in the EGF family and ErbB1-4 receptors in patients with schizophrenia [51]. We found that ErbB1 receptor levels are elevated specifically in the forebrain, whereas EGF content is reduced in the blood of patients with schizophrenia. The reduction in blood EGF concentrations is confirmed by later reports [52,53], although there is controversy still remaining [54]. In the same year of 2002, Decode Genetics Inc. found a genetic association between a neuregulin-1 haplotype and this illness by analyzing the national genome bank of Iceland [55]. The genetic association with neuregulin-1 is confirmed by later meta-analyses as well [56]. Groups in Finland reported positive interactions with the single-nucleotide polymorphism (SNP) of the EGF genome [57,58], but the other group failed to detect this genetic association in a Japanese population [59]. Groenestege et al. (2007) found that one family lineage of the EGFR-mutation-driven renal disease carries schizophrenia and other psychiatric diseases [60]. In addition to the above pioneering genetic study on neuregulin-1, the neuropathological association of neuregulin-1 with schizophrenia has also been investigated in postmortem brain and blood samples; Neuregulin-1 mRNA and its receptor protein ErbB4 are elevated in the brain [61,62,63]. Neuregulin-1 protein levels in the blood are increased in patients [64]. Those clinical studies on schizophrenia were followed by animal modeling and its translational studies [65].

In addition to the genetic hypothesis for schizophrenia, many epidemiological studies have raised various types of environmental hypotheses for schizophrenia, such as the immune–inflammatory hypothesis [31,66,67,68]. Among these, we tested the immune–inflammatory hypothesis to produce animal models for schizophrenia; prenatal or perinatal inflammatory cytokines perturb normal brain development and/or circuit organization, leading to abnormal neurocognition and emotions in schizophrenia [29,30,31,69,70]. Accordingly, we tested this hypothesis at the molecular level, challenging pregnant mice and neonatal rats with various inflammatory cytokines [71]. Other groups have tested the impact of fetal inflammation in pregnant mice, treating them with polyinosinic–polycytidylic acid (poly I:C) or lipopolysaccharide (LPS) [69,72,73], ligands of the Toll-like receptors. Of note, it is suggested that the maternal infection model established with poly I:C also involves abnormal ErbB signaling [33]. A pathological role of interleukin-6 (IL-6) is implicated in their behavioral deficits of the poly I:C-injection model, but we failed to induce behavioral impairments in their offspring with maternal and neonatal direct challenges of IL-6 [74,75]. In our cytokine challenge procedure, maternal administration of IL-2 produced the most prominent impact on offspring behaviors. In the neonatal treatment protocol, IL-1, neuregulin-1, and EGFR/ErbB1 ligands (EGF, TGFα, and epiregulin) exhibited the most remarkable effects on post-pubertal behaviors and cognitions [76,77,78,79].

Considering the strength of the behavioral impact and its reproducibility, we selected EGF and neuregulin-1 and characterized the animal models established by these cytokines. After publishing several results from the neuregulin-1 model, however, we ceased the direction of the neuregulin study and put our primary focus on the EGF animal model for the following reasons. EGF model rats or mice exhibit almost all the behavioral endophenotypes relevant to schizophrenia, including prepulse inhibition, social interaction, sensitivity to psychostimulants, and latent inhibition of fear learning, with high reproducibility [77,78,79,80,81,82,83]. In primate modeling, moreover, the neonatal EGF treatment of cynomolgus monkeys resulted in the post-pubertal emergence of behavioral abnormality [84]. A monkey challenged with EGF displayed visual hallucination-like behaviors and self-injury after five years of age. In contrast to the EGF model, one of the neuregulin-1 mouse models exhibited various face validities at the behavioral level but carried severe hearing deficits [79,80]. Thus, we expected future technical difficulty to distinguish direct and indirect effects of the hearing deficits on their cognitive functions.

In addition, our latest studies reveal that the EGF rat model exhibits most of the pathophysiological changes reported in patients with schizophrenia; electroencephalography abnormalities in duration mismatch negativity (MMN), frequency MMN, auditory steady-state response (ASSR), P300, auditory brain stem response (ABR), and functional inter-cortical connectivity [85,86,87]. In the following chapters, we summarize the dopaminergic role in the behavioral impairment of the EGF model and discuss the neuropathological implication of this model in the dopamine hypothesis for schizophrenia. As most of the above pathophysiological changes of the EGF models do not involve the dopaminergic system, we do not discuss their pathophysiology in this review.

## 2. Method for Literature Selection

We searched for previous publications using the keywords of our main topics {EGF, schizophrenia, rat/mice} and {EGF, schizophrenia, patients} in the NCBI PubMed database. The former search and the subsequent re-confirmation of their contents resulted in 38 papers, including 30 papers from our laboratory. Accordingly, the main logical flow of this review was constructed on the basis of our previous studies. In addition, clinical and genetic studies regarding EGF and schizophrenia total 14 papers, the contents of which are often confirmative, and only representative papers are discussed in this review. The other search was performed with the keywords {EGF/neuregulin, dopamine neuron}, resulting in 32 papers. Nineteen papers fitting the present discussion were adopted. To explain the backgrounds of technical terms and hypotheses, we additionally cited the literature relevant to the present discussions. Therefore, there is a limitation of our data explanation and discussions in this review. The present arguments are also hypothetical at this stage with the given uncertainty of the schizophrenia neuropathology. In this respect, this article is not a systemic review but a hypothesis-driven review.

## 3. Neurotrophic Functions of EGF and Neuregulins in Dopaminergic Neurons

### 3.1. Distributions of ErbB1 (EGFR) and ErbB4 in the Midbrain

The EGF receptor ErbB1 is distributed in dopaminergic neurons in the substantia nigra compacta, as well as in the ventral tegmental area [45,48]. Similarly, the neuregulin receptor ErbB4 is expressed in the same regions but not always in the same dopaminergic cell population [45,48]. Here, we discuss the neurotrophic actions of EGF and neuregulins on dopaminergic neurons located in these regions.

In culture, EGF enhances cell survival and neurite extension of dopaminergic neurons [48]. The neurotrophic activity of EGF is inhibited by EGF-neutralizing antibodies and by tyrosine kinase inhibitors for ErbB1 [48]. However, the neurotrophic activity of EGF was not blocked by glial cell line-derived neurotrophic factor (GDNF)-neutralizing antibody or an inhibitor of neurotrophin (TrkB1-Fc), suggesting a direct action of EGF on dopaminergic neurons. In vivo administration of ErbB1 kinase inhibitors attenuates postnatal elevation of tyrosine hydroxylase, verifying the neurotrophic role of endogenous EGF signaling in dopaminergic development [48].

GDNF is the most well-known factor for its neurotrophic action on dopaminergic neurons. This factor can prevent dopaminergic neurodegeneration in the animal models of Parkinson’s disease, and this GDNF action is mimicked by EGF [88,89,90,91,92,93]. Both factors are provided by striatal cells as retrograde factors that maintain dopaminergic neurons [94,95]. Neuregulin-1 also exerts similar neurotrophic activity in dopaminergic neurons in vivo [96,97]. However, knockout mice deficient of the ErbB4 gene exhibit normal neuroanatomy of dopamine neurons in the midbrain [98], although several controversies remain (see below).

Receptors for EGF and neuregulins, ErbB1 and ErbB4, are also expressed in several types of GABAergic populations, although this topic is not discussed in the present review paper. However, we need to point out the fact that the action of EGF on this cell population is opposite to that of dopaminergic neurons; it attenuates postnatal development or maturation of GABAergic neurons [99]. This contrasts with the positive neurotrophic actions of neuregulin-1 commonly found in the GABAergic cell populations [100,101,102].

### 3.2. Influences of EGF/Neuregulin Signaling on Dopamine Metabolism

In addition to the developmental action of EGF and neuregulins, these factors can exert functional effects on mature dopaminergic neurons in the adult stage. In vivo infusion of neuregulin-1 activates ErbB4 receptors in the prefrontal cortex, hippocampus, and dorsal striatum and elevates dopamine concentrations [103,104]. In addition, neuregulin/ErbB signaling plays regulatory roles in the expression of dopamine transporters as well as in glutamatergic synapse plasticity [103,105,106,107]. These phenomena are consistent with our previous observation of the neuregulin-1 model established by its neonatal challenges; neonatal treatment with neuregulin-1 elevates dopamine synthesis and release [79]. However, other studies on ErbB4 knockout mice have provided controversial results: the signal blockade of neuregulin-1/ErbB4 elevates striatal dopamine levels [108,109]. This appears to be in accordance with the report that the gene ablation of HB-EGF, a ligand for ErbB1/B4 receptors, resulted in the abnormal behaviors relevant to the dopaminergic system or schizophrenia pathology [110]. Therefore, the neuregulin/ErbB4 functions in dopaminergic regulation differ significantly among target cell populations, their developmental stages, or neuregulin-splicing variants [65,111].

In contrast to the physiological actions of neuregulins, the action of EGF on mature dopaminergic cells is relatively consistent across reports. EGF enhances ATP-triggered dopamine release from cultured PC12 cells [112]. In vivo administration of EGF, HB-EGF, or neuroegulin-1 elevates the concentrations of dopamine and its metabolite, 4-dihydroxyphenylacetic acid (DOPAC), in the striatum of Parkinson’s model animals [97,103,113] as well as in normal rats [114]. In a critical sense, however, it is uncertain whether the EGF-induced dopamine release is ascribed to enhanced dopamine synthesis or elevated vesicular release of this neurotransmitter.

We prepared rodent models of schizophrenia by subcutaneously injecting EGF or neuregulin-1 into neonatal rats and mice [77,78,79]. There was a marked increase in tyrosine hydroxylase in the whole forebrain regions during the postnatal administration of these factors [77,79]. Thus, the increased brain concentrations of dopamine and its metabolites presumably reflect the elevation of dopamine synthesis. At the adult stage following neonatal EGF treatment, however, we detected abnormal hyperdopaminergic innervation and higher dopamine release only in the globus pallidus. We speculate that the reason for the persistent hyperdopaminergic innervation to the globus pallidus is that the lateral area (tier) of the substantia nigra compacta, which mainly innervates the globus pallidus, is enriched with ErbB1/EGFR even at the adult stage [115].

In contrast to EGF model rats, schizophrenia model mice established by postnatal treatment with neuregulin-1 display a variety of behavioral abnormalities depending on the neuregulin-1 splicing variants administered [79,80]. In the model established by a full form of neuregulin-1β1, prefrontal hyperinnervation of A10 dopaminergic fibers is apparent, and dopamine levels are found elevated in the forebrain regions [79]. These neuregulin model mice show behavioral deficits relevant to schizophrenia. However, this mouse model exhibits a marked difference in acoustic hearing ability from the model established by another neuregulin-1 variant (a EGF core domain of neuregulin-1β) [80]. Behavioral responses to the NMDA receptor blocker MK801 also differed between the models established by two neuregulin-1 variants [65]. With the given difference among neuregulin-1 variants, the biological and behavioral effects of individual neuregulin-1 variants remain to be distinguished carefully.

### 3.3. Post-Pubertal Elevation of Dopaminergic Activity in the EGF/Neuregulin Models

**Figure 3:**
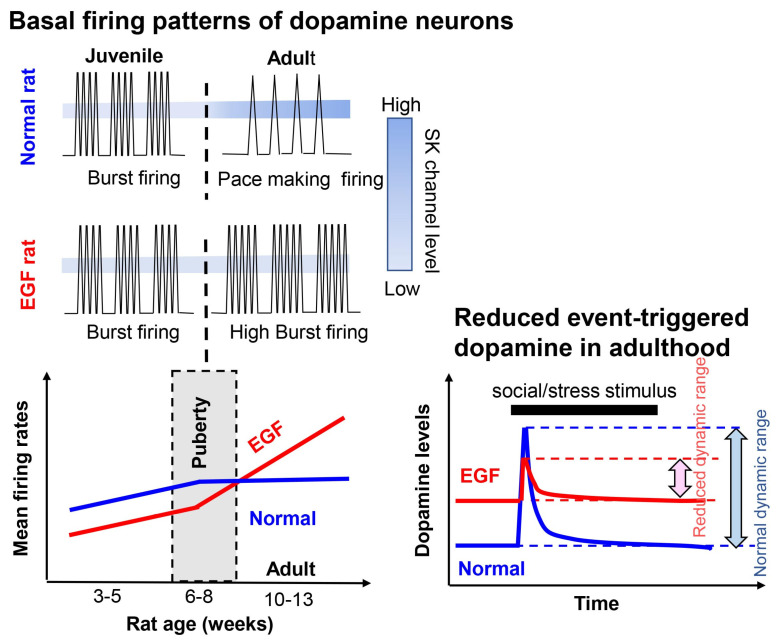
Age-dependent alteration of firing activity of dopamine neurons. Baseline burst firing is reduced in the post-pubertal stage in parallel with the increase in SK channel expression in normal development. In contrast to the profile of normal animals, baseline burst firing is elevated and the expression of SK channels is reduced in the EGF model at their post-pubertal stage [117]. Accordingly, event-triggered and stress-evoked fractions of dopamine (DA) release are relatively suppressed with the given high baseline release and might be implicated in their stress vulnerability.

One of the peculiar features of schizophrenia is that disease onset is limited to the pubertal and post-pubertal stages. Does this phenomenon correlate with dopaminergic abnormalities suggested in the EGF model? We examined the developmental alterations in the firing frequency and bursting ratio of midbrain dopaminergic neurons in EGF model rats and mice [116,117] (Figure 3). In general, midbrain dopaminergic activity and its synaptic innervation peak around puberty [118,119]. Before puberty, the firing frequency of the EGF model mice was indistinguishable or lower than that of control mice, whereas the frequency continued to increase and became higher after puberty. The burst ratio of dopaminergic firing subsided in control mice but not in EGF model mice after puberty. These phenomena agree with our pharmacological finding that SK (small conductance calcium-activated potassium) channel activity and sensitivity to apamin, implicated in the regulation of their burst firing, are reduced in EGF model mice only at the adult stage [116,117,118]. In addition, we speculate that the normal development of the local inhibitory system for dopamine neurons might also be impaired in this EGF model. In the neuregulin-1 mouse model, the attenuation factor has been identified as GABAergic inhibition from substantia nigra reticulata [120]. Disinhibition of dopaminergic neurons results in enhanced burst firing in the adult stage. Thus, almost all behavioral alterations in these models in mice, rats, and monkeys coincide with their post-pubertal abnormality of dopaminergic firing. In other animal models for schizophrenia, this coincidence is controversial among the reports. The neonatal ischemic model shows a similar increase in dopamine metabolism in the adult stage [121]. Similar abnormalities in the dopamine system or function are implicated in other animal models for schizophrenia, the prenatal methylazoxymethanol acetate (MAM) injection model and the prenatal poly I:C injection model [122,123]. More elaborate analyses regarding the temporal correlation between dopaminergic activity and behavioral alterations must be performed in these models to illustrate their association with the post-pubertal onset of schizophrenia.

## 4. Chronic Hyper-Dopaminergic Influences on Animal Models and Patients with Schizophrenia

As described above, EGF models exhibit hyperactivity of midbrain dopaminergic neurons in the substantia nigra compacta (the origin of the A9 pathway) and the ventral tegmental area (the origin of the A10 pathway) after puberty. In agreement with this, dopaminergic concentrations are elevated in the globus pallidus and medial prefrontal cortex—the target regions of these pathways. We investigated the behavioral impact of these hyperdopaminergic states in each pathway, distinguishing the resting and the stress-loaded conditions.

### 4.1. Interaction of A10 Dopaminergic Activity with Acoustic Prepulse Inhibition

**Figure 4:**
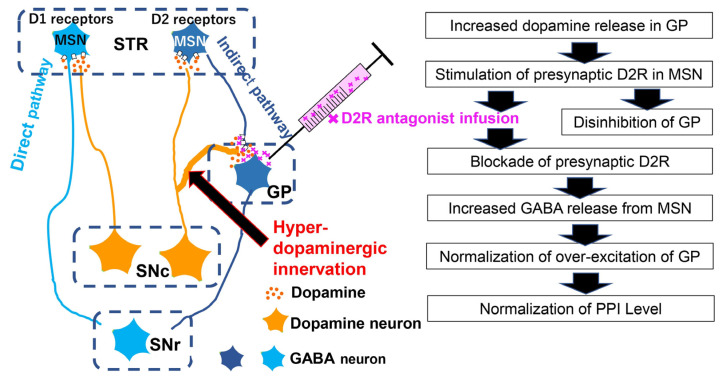
Abnormal basal ganglia circuits of the EGF model. In the EGF model, the globus pallidus receives hyper-dopamine innervation of the A9 pathway. Blockade of dopamine D2 receptors in the globus pallidus normalizes the local dopamine transmission and elevates GABA release from the terminals of medium spiny neurons (MSN). The following enhanced inhibition of GP neurons ameliorates the deficit in prepulse inhibition (PPI) [83]. STR: striatum; GP: globus pallidus; SNc: substantia nigra pars compacta; SNr: substantia nigra pars reticulata. Note; the two major circuits of direct and indirect pathways are present in the basal ganglia, counteractively regulating movement, attention, sensory filtering, etc. The direct and indirect pathways stem from the two types of MSN in STR which express dopamine D1 and D2 receptors, respectively.

The dopamine releasers of amphetamine and cocaine are known to decrease prepulse inhibition (PPI) of acoustic startle responses [124,125]. PPI reduction is induced by the non-selective dopamine receptor agonist apomorphine as well [126,127]. These reports indicated that hyperdopaminergic states are involved in the PPI reduction. Among the hyperdopaminergic regions in EGF model rats, our pharmacological interventions revealed that the globus pallidus plays a crucial role in PPI reduction [83] (Figure 4). The local infusion of a D2 receptor agonist, but not a D1 receptor agonist, into the globus pallidus of normal rats mimics the effects of EGF, that is, PPI reduction. Conversely, the local infusion of a D2 receptor antagonist, but not a D1 receptor antagonist, into the globus pallidus of EGF model rats ameliorated their PPI deficits [83]. The role of dopamine D2 receptor signaling in PPI regulation is also supported by previous pharmacological studies [128,129,130] and by gene ablation experiments of dopamine receptors [131]. However, in addition to our argument, the A10 dopaminergic pathway for the nucleus accumbens and the A9 pathway for the anteromedial striatum are also implicated in PPI regulation [132,133,134,135,136]. In these respects, controversies remain regarding the main dopamine pathway for PPI regulation and the mutual interactions of individual basal ganglia circuits.

### 4.2. Chronic Hyper-Dopaminergic States Impair Social Interaction

Antipsychotic treatment of EGF model rats with risperidone, but not with haloperidol, normalizes the dopaminergic hyperactivity of the A9 and A10 pathways [83] and their social interaction [137]. However, the pharmacological intervention of the A9 pathway does not affect the social interaction impairment of this model [83], suggesting that the basal hyperactivity of the A10 pathway stemming from the ventral tegmental is involved in this deficit.

**Figure 5:**
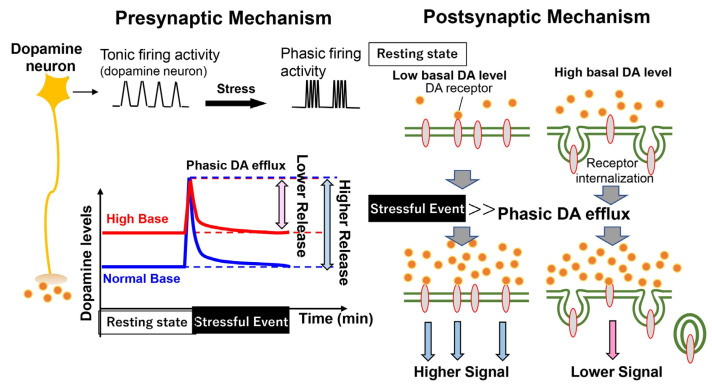
The effect of basal dopamine release on behavioral regulation. Releases of dopamine (DA) are regulated by two independent modes: tonic and phasic activities of dopamine neurons. In the resting state (a baseline condition), tonic activity contributes to maintaining basal dopamine levels in the targets. Stressful events cause a phasic activity to trigger dopamine release and acutely increase dopamine levels. In contrast, a higher basal dopamine level at the resting state decreases the dynamic range of phasic dopamine efflux, attenuating the dopamine-dependent behavioral responses (Presynaptic Mechanism). Alternatively, the increased basal dopamine level causes the internalization of dopamine receptors in the targets and downregulates the receptor signaling. Even if the event-related phasic dopamine efflux occurs, the responses of the postsynaptic dopamine receptors are attenuated (Postsynaptic Mechanism).

We tested the role of the A10 pathway by chronically or acutely manipulating the firing activity of A10 dopaminergic neurons with the pharmacogenetics tool DREADD (Designer Receptors Exclusively Activated by Designer Drugs) [137,138]. DREADD-driven artificial normalization (i.e., a decrease) of A10 dopaminergic activity in EGF model rats ameliorates their social deficits [137]. Conversely, chronic upregulation of A10 dopaminergic activity in normal rats impairs social interaction [138]. Chronic dopamine elevation at the basal state negatively affects subsequent event-related dopamine responses and functions with two potential mechanisms (Figure 5). Our results suggest that the chronic and acute release of dopamine display opposite actions on the target functions. Chronic dopamine elevation at the basal state negatively affects subsequent dopamine responses and/functions. One of the molecular mechanisms is that with the higher baseline release of dopamine, the event-triggered fraction (i.e., the dynamic range) of dopamine release becomes lower. The other is that chronic dopamine release at the basal state induces the dopamine receptor internalization and the downregulation of receptor signaling, resulting in the attenuation of postsynaptic responses [139,140]. Therefore, acute stimulation of A10 dopaminergic neurons promotes social interactions [138,141]. Conversely, acute optogenetic suppression of A10 dopaminergic activity results in social interaction deficits [142]. In this respect, the event-triggered fraction (i.e., the dynamic range) of dopamine release determines the magnitude of event-related dopamine function and response. This argument suggests the possibility that dopaminergic hyperactivity at the resting state can be involved in parts of the negative symptoms of schizophrenia, such as stress vulnerability and anhedonia. A similar chronic abnormality in the A10 pathway would be implicated in social abnormalities in other animal models for schizophrenia, such as a chronic stress model with social defeats, a MAM-induced model, and a ventral hippocampus lesion model [122,143]. However, the antipsychotic responses of these models differ significantly [122,144].

In spite of the above argument, the medication effects of conventional antipsychotic drugs is limited on the negative symptoms of patients with schizophrenia [145,146,147,148]. Additional mechanisms would be required to fully illustrate the persistent nature of the depression-like negative symptoms in schizophrenia and in these animal models. Although the neuropathologic underpinnings of schizophrenia and depression are controversial, one of the potential explanations for the social deficits of the model animals would represent the neurodegenerative and cytotoxic actions of dopamine or inflammatory cytokines [149,150,151,152,153,154].

In general, the dopamine hypothesis for schizophrenia fails to illustrate the negative symptoms of this illness [145]. One is that typical antipsychotic drugs, which target dopamine D2 receptors, are ineffective against the negative symptoms of social withdrawal and anhedonia [145,146,147,148]. The chronic abuse of amphetamine or cocaine does not result in negative symptoms. However, the latest brain imaging studies have supported the dopamine hypothesis. Several PET studies have consistently reported a reduction in dopamine D1 receptor occupancy in the prefrontal cortex of patients with schizophrenia [155,156,157,158]. These studies also pointed out a negative correlation between occupancies and negative symptom scales across patients [155]. This reduction in dopamine receptor occupancy can be ascribed to either a decrease in receptor density or an increase in dopamine release. The latter explanation is supported by the following two findings. Incorporation of L-(beta 11C)-DOPA into the prefrontal cortex is elevated in patients with schizophrenia compared with that of control subjects [159]. A side product of dopamine synthesis, melanin, is highly deposited in the substantia nigra regions of patients with schizophrenia [160,161]. Although this explanation is not discrepant with our results from the EGF model, it would be better to verify whether the reduction in dopamine D1 receptor occupancy indeed stems from chronic upregulation of dopamine release.

## 5. Drug Development Targeting EGF/ErbB Signals 

According to our initial postmortem findings in schizophrenia patients, ErbB1 receptors are upregulated, and EGF contents are downregulated in the forebrain regions of the patients [51]. This result suggests that abnormal EGF/ErbB1 signaling persists throughout life. However, it is unclear whether EGF/ErbB1 signals are enhanced or reduced in patients with schizophrenia. We attempted to answer this question using animal models. We chronically administered EGF into the ventricle of adult rat brains and monitored behavioral alterations in prepulse inhibition and social interaction [114]. Even in the adult stage, EGF infusion increases dopamine metabolism and PPI deficits [114]. This finding suggests the possibility that EGF model rats, established by perinatal EGF treatment, continue to harbor abnormal hyper-EGF/ErbB1 signaling in the brain.

To test this hypothesis, we administered specific inhibitors of EGF receptor kinase (i.e., gefitinib, erlotinib, and PD153035) to the lateral ventricle of EGF model rats [162]. These ErbB1 kinase inhibitors ameliorate the deficits in PPI and latent inhibition but not the social interactions of this model (Table 1). In parallel, the firing activity of midbrain dopaminergic neurons is normalized [162]. The antipsychotic action of these ErbB kinase inhibitors is not limited to the EGF model rats. These inhibitors also ameliorate several behavioral endophenotypes in rat models established by neonatal hippocampal lesioning [163]. Tadmor et al. (2018) tested another quinazoline ErbB inhibitor, JNJ28871063, and reported that this inhibitor ameliorates the phencyclidine (PCP)-induced decrease in social interaction, which is implicated in the negative symptoms of schizophrenia [164]. However, these ErbB kinase inhibitors have a limited capability to penetrate the blood–brain barrier. Alternatively, we discovered a compound that inhibits ErbB kinases and penetrates the blood–brain barrier: emodin [165]. Peripheral administration of emodin reduces PPI deficits and latent inhibition abnormalities in the EGF model.

Despite their antipsychotic potential, ErbB kinase inhibitors in the quinazoline family, which are prescribed to patients with cancer, are known to exert severe side effects such as interstitial pneumonia [167]. In this respect, it is challenging to develop new antipsychotic drugs targeting ErbB receptors (Table 1).

We also explored the possibility that the current antipsychotic drugs attenuate ErbB receptor kinases and contribute to their pharmacological profiles. Among many antipsychotic drugs, clozapine is known to be a unique atypical antipsychotic which exhibits antipsychotic efficacy on drug-tolerant patients with schizophrenia as well as on their negative symptoms and cognitive decline [168,169]. However, its prescription is strictly controlled, as clozapine often shows a severe side effect of agranulocytosis [168]. How does clozapine produce those favorable and unfavorable actions? We hypothesized that clozapine involves ErbB receptor signaling. To address this question, we exposed cultured brain tumor cells to low doses of clozapine and EGF [166]. Clozapine attenuated EGF-triggered growth and survival of tumor cells, similar to ErbB kinase inhibitors. To characterize the molecular interaction between clozapine and ErbB kinases, we tested the in vitro activity of clozapine using pure recombinant ErbB1-4 kinases. Tyrosine kinase activities of ErbB1-4 receptors were suppressed in vitro by sub-micromolar ranges of clozapine without any other components, indicating that clozapine directly acts on ErbB kinases and blocks their enzyme activity. These results indicate that the unique action of clozapine involves ErbB kinase inhibition, although several ambiguities and controversies still remain [170,171].

## 6. Provisional Conclusion

The members of the EGF family include a large variety of growth factors and cytokines expressed in the CNS, regulating dopaminergic development and functions. Their signal transduction is tightly connected to the immune inflammatory signaling stemming from TLRs. Accordingly, perinatal and prenatal infection and inflammation stimulate the TLR system to induce EGF/ErbB hyper-signaling, leading to abnormal dopamine firing and their phenotypic development/connections. Of note, the dopamine dysfunction of our EGF models reaches the peak at the post-pubertal stage. In particular, chronic hyperdopaminergic states at the adult resting state produce a negative impact on the event-triggered dopamine release and responses which are implicated in motivation and stress resilience.

## 7. Future Directions

Including the EGF family, various inflammatory cytokines and their signals are implicated in the molecular pathology of negative symptoms and/or depression [31,36,66,67,68]. A large variety of effective anti-inflammatory drugs have been developed, and some of those would be effective to ameliorate those psychiatric symptoms. These drugs mainly consist of small molecules and immunoglobulins targeting cytokine receptors and their signaling molecules [172]. In addition, the molecular structures of the ligand–ErbB receptor complex have been elucidated, which should hint at a new drug design targeting these receptors [173,174]. We hope that the present review will help future diversion of the pre-existing anti-inflammatory or anti-ErbB drugs as well as the development of novel antipsychotic drugs targeting these signal pathways.

## Figures and Tables

**Table 1:** Antipsychotic-like effects of drugs acting on ErbB receptor kinases.

Drug	Target	Assay Model	Effects	References
gefitinib	ErbB1	EGF, VHL	PPI, LI	[162,163]
PD153035	ErbB1/B2	EGF, VHL	PPI	[162,163]
erlotinib	ErbB1	VHL	PPI	[163]
JNJ28871063	pan-ErbB	PCP	Social Interaction	[164]
emodin	tyrosine kinases	EGF, VHL	PPI, LI	[165]
clozapine	pan-ErbB	in vitro kinase	Tyr phosphorylation	[166]

Antipsychotic-like activities of the agents inhibiting ErbB receptor kinases are tested in various animal models for schizophrenia or the in vitro system. Abbreviations used: EGF: EGF model established by its perinatal challenges, VHL: a schizophrenia model established by ventral hippocampal lesioning, PCP: a model established by sub-chronic phencyclidine administration, PPI: prepulse inhibition, LI: latent inhibition of fear learning.

## Data Availability

Not applicable.

## Abbreviations

EGF: epidermal growth factor; TLR: Toll-like receptor; NGF: nerve growth factor; HB-EGF: heparin-binding EGF-like growth factor; TGFα: transforming growth factor alpha; CNS: central nervous system; VGF: vaccinia virus growth factor; CGF: cowpox growth factor; SPGF: smallpox growth factor; SFGF: shope fibroma virus growth factor; MGF: myxoma virus growth factor; PKC: protein kinase C; ROS: reactive oxygen species; ADAM: a disintegrin and metalloprotease; LPA: lysophosphatidic acid; NOX: NADPH oxidase; GPCR: G-protein-coupled receptor; SNP: single-nucleotide polymorphism; poly I:C: polyinosinic–polycytidylic acid; LPS: lipopolysaccharide; IL: interleukin; ASSR: auditory steady-state response; ABR: auditory brain stem response; MMN: mismatch negativity; GDNF: glial cell line-derived neurotrophic factor; DOPAC: 4-dihydroxyphenylacetic acid; SK: small conductance calcium-activated potassium; MAM: methylazoxymethanol acetate; DA: dopamine; DOPA: L-3,4-dihydroxyphenylalanine; PPI: prepulse inhibition; STR; striatum; GP: globus pallidus; MSN: medium spiny neurons; SNc: substantia nigra pars compacta; SNr: substantia nigra pars reticulata; DREADD: designer receptors exclusively activated by designer drugs; CNO: clozapine-N-oxide; PCP: phencyclidine; LI: latent inhibition; VHL: ventral hippocampal lesion.
